# Effects of Drying Methods on Chemical Composition, Lipid Oxidation, and Fatty Acid Profile of a Traditional Dried Meat Kaddid

**DOI:** 10.3390/foods12203837

**Published:** 2023-10-20

**Authors:** Amira Zioud, Wafa Hajji, Sandra Lobón, Margalida Joy, Juan R. Bertolin, Samir Smeti, Meriem Chabbouh, Sihem Bellagha, Ines Essid

**Affiliations:** 1 UR-PATIO (UR17AGR01), National Institute of Agronomy of Tunisia (INAT), University of Carthage, 43 Avenue Charles Nicole, Tunis 1082, Tunisia; wafa.hajji.new@gmail.com (W.H.); meriem.chabbouh@inat.ucar.tn (M.C.); sihem.bellagha@inat.ucar.tn (S.B.); ines.essid@inat.ucar.tn (I.E.); 2 AgriFood Research and Technology Centre of Aragon (CITA-Aragón), The AgriFood Institute of Aragón-IA2, CITA-University of Zaragoza, Avda, Montañana, 50059 Zaragoza, Spain; slobon@cita-aragon.es (S.L.); mjoy@cita-aragon.es (M.J.); jrbertolin@cita-aragon.es (J.R.B.); 3 Laboratoire de Productions Animales et Fourragères, INRA-Tunisia, University of Carthage, rue Hédi Karray, Ariana 2049, Tunisia; sam_fsb@live.fr

**Keywords:** drying, innovation, kaddid, oxidation, fatty acid

## Abstract

This work aimed to study the effect of three drying methods, namely sun-drying (SD) (T = 32 °C), continuous convective drying (CCD) (T = 35 ± 2 °C) and interval starting accessibility Drying (ISAD) (T = 35 ± 2 °C) with an active time of 30 s (t_on_) and a tempering time of 60 s (t_off_), on selected quality characteristics of a traditional dry-salted meat product known as “kaddid”. The analyses of chemical composition, lipid oxidation and fatty acid profile of kaddid were carried out before and after 45 days of storage (t = 0 vs. t = 45) at ambient temperature. Chemical composition and lipid oxidation (TBARS) of kaddid were affected by the drying methods. The CCD samples showed the lowest level of lipid oxidation. Protein content was better preserved via the ISAD method (7.27 g/kg DM). The fatty acid profile revealed the lowest mono-unsaturated fatty acid content in the ISAD samples; however, no significant difference was observed between the drying processes for the total poly-unsaturated fatty acid content. The storage period led to a significant decrease in the SFA values of CCD and ISAD samples against an increase in the MUFA ones. ISAD appeared to be a promising drying mode with a lower effective drying time and a good product quality preservation.

## 1. Introduction

Numerous ancient preservation techniques are used in traditional meat processing, such as salting, curing, drying, heating, and fermentation. Among these methods, the most commons remain salting and drying, which aims to prevent spoilage and to prolong the life of the meat over a long period and at ambient temperature [1,2]. Indeed, in the absence of a reliable cold chain, meat drying remains the most convenient option for meat preservation and storage in developing countries with hot and humid climates [3].

Kaddid is a well-known traditional product in Maghreb countries. It is salted, seasoned, and dried beef or lamb. This product has retained a traditional preparation process, which is not only different between regions but also poorly controlled and can be exposed to insect infestation and microbial contamination during the sun-drying step [4]. On the other hand, the industrialization of this product and the control of its drying process using conventional methods has many disadvantages, mainly energy consumption. Convection drying is one of the most energy-intensive steps in food processing and constitutes up to 15% of the industry’s total energy consumption [5]. Furthermore, the processing and drying conditions of meat products affect their physicochemical, sensory, and nutritional properties. Indeed, both conventional and innovative drying processes frequently lead to overheating of food products, which results in denaturation of nutrients and degradation of sensory properties [6]. Additionally, the fatty acid composition of meat products influences their stability and nutritional quality. Wazir [7] reported that a higher polyunsaturated fatty acid (PUFA) content accelerates the occurrence of secondary lipid oxidation and then sensorial deterioration. Hence, exploring new efficient drying methods and technologies is required to provide products with improved characteristics and desirable stability while reducing energy consumption. Interval starting accessibility drying (ISAD) is an innovative drying method in which the product is subjected to very short periods of active heating (t on) interspersed with rest periods called tempering periods (t off). The redistribution of water and product temperature during tempering periods allows the meat to reach the highest water content on the surface of the product, thus reducing the moisture gradients and therefore the drying time [5].

Therefore, the present work was conducted to study the effect of three drying methods, ISAD (innovative method), CCD (conventional method), and SD (traditional method), on the chemical composition, oxidation rate, fatty acid profile, and shelf life of a Tunisian traditional meat product kaddid.

## 2. Materials and Methods

### 2.1. Sample Preparation

**Figure 1:**
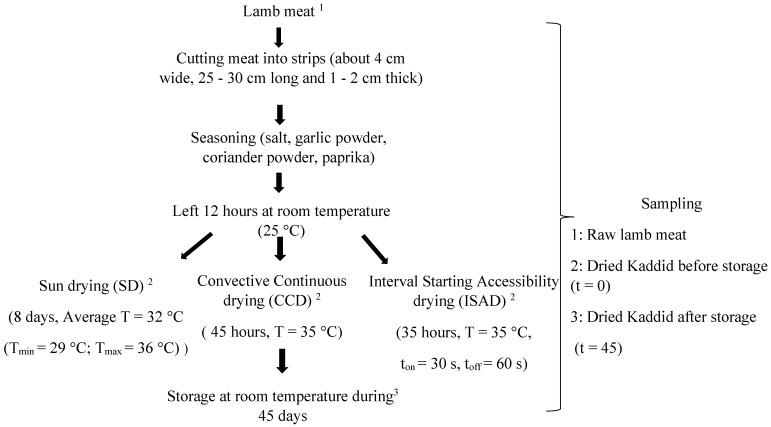
Flow chart of kaddid preparation.

Lamb meat was purchased from a local butcher in Tunisia and came from 5 lambs of the Barbarine breed (7 months old; 26 ± 1.7 kg body weight). The lambs were reared under the same feeding system. The preparation of the kaddid was carried out according to the diagram in Figure 1. Pieces of meat (15 kg) were collected from the lamb leg and cut carefully into slices. Meat samples were then seasoned and mixed well with a powdered spice mixture. The spices used were garlic powder, coriander powder, salt, and paprika in the quantities of 4 g, 6 g, 1.5 g, and 0.5 g/100 g of meat, respectively. The meat was then kept at room temperature (25 °C) for 12 h. After seasoning, the slices were randomly divided into 3 homogeneous batches of equal weight (5 kg). Each batch was divided into 3 samples (*n* = 3) and dried using different methods. The kaddid was then stored in plastic boxes at room temperature for 45 days.

### 2.2. Drying Methods

The drying of the kaddid was carried out using three different methods (Figure 1), namely natural sun-drying (SD), continuous convective drying (CCD), and interval convective drying (ISAD) [5]. The SD was conducted in the month of August for 8 days at an average temperature of 32 °C (T_min_ = 29 °C; T_max_ = 36 °C) and an average relative humidity of 60%. Meat strips were hung on a wire during the day with a mosquito net cover to avoid insect infestation. Samples were collected each day before sunset and stored overnight in a cool airy place until they reached a water activity (aw) of 0.57, which indicates the end of drying.

Convective drying was carried out in an oven (SHEL LAB model 1375 FX, Cornelius, OR 97113, USA) at a temperature of 35 ± 2 °C over a period of 45 h. The oven was preheated to the selected drying temperature for approximately 30 min before the samples were placed in the oven to achieve stable conditions [8]. Meat samples were spread out on a pierced tray that allowed for air circulation during the drying process. Drying was completed when the water activity reached 0.57.

The interval starting accessibility drying (ISAD) technique is an innovative drying method that consists of drying for very short durations (t_on_) intersected by tempering times (t_off_) [5]. The drying was performed over 35 h with an airflow velocity of 3 m·s^−1^ at a temperature of 35 ± 2 °C and with t_on_ = 30 s and t_off_ = 60 s until a water activity of 0.57 was reached.

The ISAD drying method was significant in terms of drying time. This method reduced the effective drying time by 22% compared to CCD to achieve the same final water activity.

After drying, all samples of kaddid were stored at room temperature in a closed plastic box for 45 days (t = 45).

### 2.3. Chemical Analyses

The biochemical analyses were performed on raw lamb meat (RM), kaddid samples (SD, CCD and ISAD kaddid) after drying (t = 0), and after 45 days of storage at room temperature (t = 45). To assess the chemical composition, tocopherols, retinol, and fatty acids, samples of meat and kaddid were freeze-dried to obtain dry matter (DM).

#### 2.3.1. Protein Content

Crude protein content (CP) was analyzed using a nitrogen analyzer (model NA 2100, CE Instruments, Thermoquest SA, Barcelona, Spain) according to the Dumas method [9]. A sample of known mass was burned at a temperature between 800 and 900 °C and in the presence of a CuO + Pt/Al_2_O_3_ catalyst. During the reaction, carbon dioxide, water, and nitrogen oxides were produced. The reduction of the nitrogen oxide to nitrogen gas (N_2_) was carried out by means of copper wire. The nitrogen-measuring instrument was first calibrated with pure methionine with a known nitrogen concentration. The signal measured by the thermal conductivity detector for the sample could then be converted to nitrogen content.

The amount of N_2_ detected is expressed in milligrams. Protein content (g/kg) was calculated with the weight of the sample and an N_2_-protein conversion factor (6.25).

#### 2.3.2. Total Fat Content

Total fat was measured via acid hydrolysis in filter bags using the XT10 Ankom extractor (Ankom Technology Corporation, New York, NY, USA) [10]. A 1 g sample of ground freeze-dried meat was placed in labeled filter bags and sealed with a heat sealer. After drying in the oven for 24 h, the weighed filter bags were inserted into a multi-bag holder and covered with petroleum ether. The sample fat content was then susceptible to extraction via the solvent. The amount of fat expressed in % was determined from the difference in mass.

#### 2.3.3. Lipid Oxidation

The analyses of the lipid oxidation of meat and kaddid were carried out following the method of thiobarbituric acid reactive substance (TBARS) as described by Botsoglou [11] with modifications. In order to remove the spices covering the surface of the kaddid, the samples were well cleaned with a dry brush. Since the prepared kaddid samples were dried to a low water activity, their water retention capacity was high, resulting in a dry, pasty mixture. Therefore, the amount of sample used for the analysis was reduced to 5 g. A calibration curve was used to calculate the sample concentration. TBARS values were expressed as mg malondialdehyde (MDA) kg^−1^ of muscle.

#### 2.3.4. Fatty Acids

The fatty acid (FA) profile of the lamb meat and kaddid, respectively, was assessed according to the method of Lee et al. [12] with modifications. The analyses were carried out via a gas chromatograph (Bruker 436 Scion gas, Billerica, MA, USA) equipped with a cyanopropyl capillary column (BR-2560, 200 m × 0.25 mm ID × 0.20 µm thickness, Bruker, Billerica, MA, USA) with a flame ionization detector for fatty acid methyl esters (FAMEs) and Compass CDS. Relative retention times and standard references were used for the identification of FAs (GLC-532, GLC-401, GLC-643, GLC-642, GLC-463, C18:1 t11, C19:0 and C23:0 (Nu-Chek-Prep Inc., Elysian MN, USA)). After individual FA determination, the sum of the saturated fatty acids (SFA), monounsaturated FAs (MUFA), polyunsaturated FAs (PUFA) was calculated and is reported as % FAMEs.

#### 2.3.5. Tocopherols and Retinol Content

Tocopherols and retinol content were determined following the methodology described by Bertolín et al. [13]. A 0.2 g sample of freeze-dried meat was saponified with 3 mL of saponification solution (10% *w*/*v* potassium hydroxide in a 50:50 mixture of ethanol and distilled water *v*:*v*) overnight under an inert N2 atmosphere. Subsequently, the analytes were extracted twice with 5 mL of n-hexane: ethyl acetate (9:1, *v*:*v*) with 5 µg mL^−1^ of BHT mixture and evaporated in a vacuum evaporator for 30–40 min at 40 °C. A 1 Ml quantity of acetonitrile: dichloromethane: methanol (75:10:15) was added to dissolve the dry residue, then filtered through a PTFE filter into a 2 mL HPLC* vial. Tocopherols and retinol analyses were performed using an ACQUITY UPLC H-Class liquid chromatograph (Waters, Milford, MA, USA) equipped with a silica-based column (Acquity UPLC HSS T3, 1.8 µm × 2.1 mm × 150 mm column, Waters), an absorbance detector (Acquity UPLC Photodiode Array PDA eλ Detector, Waters), and a silica-based bound phase column. Tocopherols were detected by measuring the fluorescent emission at λexc = 295 nm and λemi = 330 nm and retinol by the absorbance at 325 nm. Quantification of tocopherols and retinol was performed using a five-point calibration curve from pure standards. Tocopherols and retinol values are expressed in μg/g of DM.

#### 2.3.6. Water Activity

The water activity of kaddid was measured using a pre-calibrated rotronic water activity meter (Hygro-Lab C1, Decagon Devices, Inc. Pullman, WA 99163, US) at 20 °C ± 0.5.

### 2.4. Statistical Analyses

All the measurements were conducted in triplicate. The statistical analysis of the results obtained was carried out using the SAS system for Windows 9.0. A linear–mixed model procedure (PROC MIXED, SAS, NC, USA) was used to analyze the significance of the different drying modes and storage time on TBARS, chemical composition, and FAMEs measurements at 0.05 of significance level.

The drying mode, the storage time, and their interactions were included as fixed effects, and repetition was included as a random effect. In order to find the relationship between each two response variables, a matrix of correlation was carried out between all pairs using Spearman’S correlation coefficient.

## 3. Results and Discussion

### 3.1. Chemical Composition

Table 1 presents the chemical composition results of raw meat, sun-dried, ISAD, and CCD kaddid. The protein content of raw lamb meat was found to be 8.07 g/kg of DM (Table 1). The value obtained in this study is slightly higher than the one found by Tibaoui et al. [14] and lower than the value obtained by Blanco et al. [15], which is around 9.23 g/kg of DM. The stability of a meat product is influenced by its lipid profile and muscle tissue composition which, in turn, are related to the antioxidant capacity and protein concentration [16]. Gonzales [17] reported that chemical composition of lamb meat, notably the protein content, depends on the animal breed, its origin, and the rearing conditions. Results showed a significant decrease in protein content after drying. The highest protein values were obtained via the ISAD method (7.26 g/kg of DM) followed by the CCD (6.55 g/kg of DM) (*p* = 0.0157). Gliguem et al. [18] described similar behavior while drying crabmeat using the ISAD and CCD methods. They found slightly higher protein content following ISAD. This trend was correlated with the kinetics of water loss to highlight the greater thermal degradation of proteins and the loss of their biological activity in the presence of water [18]. Several authors have reported that heat treatments such as drying may induce several changes in the protein content of food products, notably the destruction of sulfur-containing amino acids [18,19,20]. As discussed in the literature, during meat drying, protein structure is denaturized as a partial consequence of the decrease in water content [21]. Changes in chemical bonds within or between protein molecules and weakening of the intramolecular water binding strength induce the change to the protein structure [22]. This change is confirmed by Rao’s work [21] using differential scanning calorimetry after drying sheep meat at 35 °C. The range of values found for the different kaddid groups in this study was close to that found in Apata’s work on drying kilishi via different solar drying methods [23]. The storage time affected the CCD and the SD kaddid samples (*p* = 0.005) with an increase from 6.55 g/kg of DM (t = 0) to 7.34 g/kg of DM (t = 45) for the CCD samples and from 6.44 g/kg of DM (t = 0) to 6.98 g/kg of DM (t = 45), while no interaction effect with the drying mode was found (0.082).

The fat content of the lamb meat obtained in the present study was 1.28 g/kg DM. Several authors have reported that the amount and the nature of the lipids present in muscles largely depend on the breed of the animal as well as on dietary intake [24,25]. The results, summarized in Table 1, showed a significant effect of drying methods on kaddid fat content (*p* = 0.0051). The amount of fat content increased after sun-drying and CCD compared to the RM. This variation between the three drying modes tested can be attributed to the different duration of the drying process and heat exposure, i.e., 8 days for SD, 45 h for CCD, and 35 h for ISAD. According to Thippareddi and Sanchez [26], a temperature close to the melting point of the fat (37–40 °C) could be the cause of an increase in fat content detected. Fat increase may be due to either the infiltration of the melted fat into the muscle tissue or the alteration of the muscle structure by heat, which facilitates extraction of the fat from the muscle via organic solvent. The storage time significantly reduced the fat content of the kaddid samples (*p* = 0.0013). In contrast, a study carried out by Wazir et al. [7] on shredded beef and chicken products showed an increase in lipid extractability with increasing temperature and storage time and explained this tendency as being due to the extensive disruption of the meat structures at a later stage of storage, leading to the separation of the fat from samples and increasing its accessibility to the solvent.

### 3.2. Tocopherols and Retinol

The total tocopherol content of the raw meat was approximately 10.45 µg/g DM, composed of 9.74 µg/g DM α-tocopherol, 0.55 µg/g DM ϒ-tocopherol, and 0.16 µg/g DM δ-tocopherol (Table 2).

These results are consistent with those reported by Tibaoui et al. [14] for the meat of ewes following the addition of different concentrations of distilled myrtle residues. Indeed, the presence of α-tocopherol in animal and human tissues is linked to feed composition. Several studies have been conducted to improve the content of tocopherols in meat, specifically α-tocopherol, which is the most prevalent form in different animal tissues [14,16,27,28]. Kaddid samples showed an abundant amount of α-tocopherol compared to ϒ- and δ-tocopherol. There is a preference for the absorption of α-tocopherol over ϒ-tocopherol in the animal’s system [29]. The contents of α-, ϒ-, and δ-tocopherol and retinol were significantly affected by the drying method (*p* < 0.05). During drying, a significant decrease in total tocopherol content was recorded for all three treatments. Results given in Table 2 showed a higher preservation of α-tocopherol content with the CCD and ISAD treatments compared to the SD method. However, there was no significant difference between the two methods. The high loss of tocopherols during meat processing was directly related to the drying methods and their durations. Sabliov et al. [30] reported an increase in the degradation of free α-tocopherol and its rate with increasing temperature. Wang et al. [31] reported that a lengthy exposure to heat under normal atmospheric conditions and the presence of several factors such as oxygen, light, minerals, and hydroperoxides increases the photo-oxidation of α-tocopherol.

Storage time mainly affected the contents of α-tocopherol and retinol. It is worth noting that studies dealing with retinol are very limited and even rare when it comes to meat and meat products.

### 3.3. TBARS

**Figure 2:**
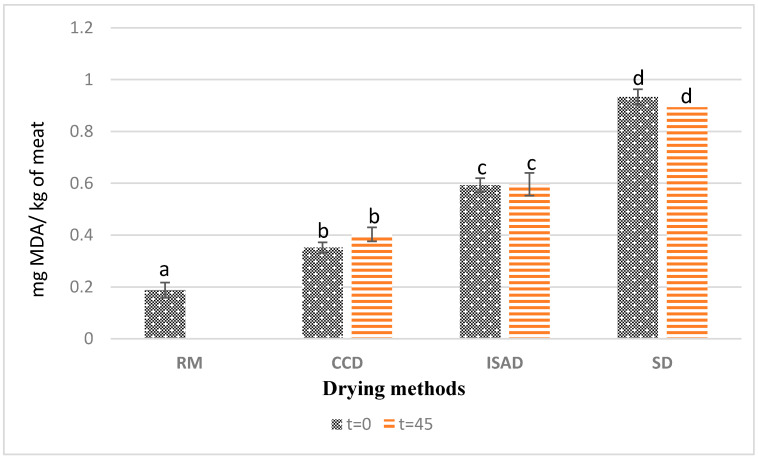
Effect of drying method and storage time on kaddid lipid oxidation (TBARS). The means with different letters differ significantly (*p* < 0.05); t = 0: after drying; t = 45: after 45 days of storage of dried kaddid; RM: raw meat; SD: sun-drying; ISAD: interval convective drying; CCD: continuous convective drying.

The results presented in Figure 2 showed a significant effect from the applied drying methods on the values of thiobarbituric acid reactive substances (TBARSs). CCD-dried kaddid showed the lowest fat oxidation rate, with 0.35 mg MDA/kg FM at t = 0, whereas the highest values were obtained via sun-drying with 0.93 mg MDA/kg FM at t = 0. The TBARS contents obtained from CCD-dried meat were lower than the values obtained by Teixeira et al. [32] for dry-cured sheep leg meat (0.52 mg MDA/Kg) and the values reported by Dorg et al. [33] for dried sheep meat. The values obtained for all treatments were below the rancidity perception threshold (2.0 mg MDA kg^−1^) and remained below the acceptability threshold of 1 mg MDA/kg recommended for lamb meat quality [34]. This low level of lipid oxidation could be explained by the presence of spices in the product, such as paprika and garlic, known for their antioxidant activity [34,35].

Several factors involved during the processing, such as exposure to oxygen and temperature, can influence the lipid oxidation level in meat and meat products [36,37]. The high TBARS value measured for the sun-dried kaddid (SD), compared to the product obtained from ISAD and CCD, can therefore be explained by the long exposure of the kaddid over 8 days of sun-drying to sunlight, heat, and oxygen. Several authors have pointed out the effect of light and exposure time on the rate of oxidation. Lorenzo et al. [38] reported that ultraviolet radiation, in the presence of sensitizers such as myoglobin, facilitated photo-oxidation, and Cooper et al. [39] demonstrated by working on beef meat that the TBARS content increased significantly with the length of exposure to light. A possible explanation for the low lipid oxidation found in ISAD and CCD kaddid might be the high amount of α-tocopherol mentioned in Table 2. According to Wang et al. [31], the antioxidant activity of α-tocopherol improves the quality of meat products by decreasing their lipid oxidation. In the same context, the findings of Tibaoui et al. [14] and Gonzalez et al. [27] confirmed the significant correlation found between TBARSs and α-tocopherol values.

In contrast, no effects of storage time were found for the three drying methods. Compared to other studies, TBARS results obtained in this work for raw meat and sun-dried kaddid were slightly lower than the range of values found by Bader et al. [40] for sun-dried lamb meat kaddid at t = 0 and after 30 and 90 days of storage in jars at room temperature (mainly varying between 15 and 25 °C). Indeed, the age and weight of the animal at the time of slaughter affect the fat content and its polyunsaturated fatty acid content, which impact lipid oxidation [41,42].

### 3.4. Fatty Acid Profile

The nutritional quality of dried meat products is highly dependent on their fatty acid composition, given the susceptibility of meat to lipid oxidation, which is largely dependent on the degree of unsaturation of the fatty acids (FAs). Table 3 and Table 4 showed the fatty acid and fatty acids group content of raw meat and kaddid samples obtained via different drying methods before and after storage.

MUFA (46.34%) and SFA (43.31%) constituted the main fatty acid groups of raw meat, followed by PUFA (10.34%). The SFA content found in this study is slightly lower than that documented in the literature (51–55%), whereas the MUFA content is higher [14,43].

With regard to each fatty acid detected in the raw lamb meat and kaddid samples, the most abundant fatty acid was oleic acid (C18:1 9 cis), as is common in such products. In the present study, it represented 30.34% of the total fatty acids for raw meat and varied between 25.72 and 34.22% for the kaddid samples. The main SFA in raw meat and processed kaddid were palmitic acid (C16:0), followed by stearic acid (C18:0) and myristic acid (C14:0).

The analysis of the intramuscular fatty acid profile revealed a significant effect of drying methods on SFA, MUFA, and on the ratio n6/n3. After drying (t = 0), the SFA of ISAD and CCD kaddid increased notably compared to fresh meat (43.31%) to 54.06% and 51.88%, respectively. Nevertheless, the MUFA content showed a decrease. The high content of SFA obtained after drying is mainly linked to the rise in C18:0 compared to the value for raw meat and the decrease of the PUFA, which means a variation in fatty acids group proportions. No significant differences were noted between the drying processes on total PUFA content or PUFA/SFA ratio, which is widely used for food and meat products to evaluate the nutritional value of human diet fat. However, the values decreased during drying for all kaddid samples. The highest PUFA value was in SD kaddid (7.42%) and the lowest in CCD kaddid (7.01%). The same variation tendency for SFA and PUFA was reported by Samples et al. [44] after drying and smoking reindeer meat compared to raw meat.

During drying, the proportion of myristic acid (C14:0), which has a potential cholesterol-raising activity and therefore promotes hypercholesterolemia, increased significantly compared to raw meat. Similarly, an increase in the proportion of stearic acid (C18:0) was identified after drying with the ISAD and CCD methods, while a significant decrease of oleic and linoleic acids was reported.

The majority of the fatty acids presented tended to differ significantly among the drying methods except for myristic acid. CCD drying revealed the lowest percentage of oleic acid (C18:1 9 cis). However, the opposite was observed for stearic acid C18:0 content (20.79% vs. 21.75%). Storage time did not significantly affect the myristic acid C14:0 (*p* > 0.05) and palmitic acid C16:0 (*p* > 0.05) contents. The percentage of stearic acid C18:0 changed after storage and was lower in the CCD kaddid than in the other two drying methods.

Most fatty acids groups and ratios were significantly affected by storage. The SFA content of ISAD and CCD kaddid showed a decrease during storage from 54.06% and 51.87% at t = 0, respectively, to 44.380% and 43.123% at t = 45, respectively, which explained the rise of the ratio PUFA/SFA for ISAD and CCD kaddid. These results can be explained by lipid hydrolysis. In dried–cured meat, high salt content and low aw contribute to lipid hydrolysis [45]. Results obtained in this study showed a PUFA percentage below 9% in all kaddid samples. The interaction between the drying process and the storage time had no significant effect on total PUFAs (*p* = 0.755).

### 3.5. Matrix of Correlation

Table 5 presents the Spearman product moment correlation coefficients between the drying mode and storage time parameters and the response variables. These coefficients assess both the statistical significance and the strength of the linear relationships between the various pairs of variables. Their values span a range from −1 (blue) to +1 (red) and indicate statistically significant non-zero correlations at the 95% confidence level.

The correlation matrix shows a significant relationship between storage time and the responses studied: in terms of α-tocopherol, IMF, proteins, and fatty acid groups, these results are in concordance with the findings previously reported in the current study. It should be noted that positive correlations were found between the different parameters, particularly for the PUFA and PUFA/SFA, as shown by the correlation coefficients > 0.8. A significant negative relationship was demonstrated between PUFA/SFA and SFA variables. This implies that as the amount of SFA content decreases during storage, the PUFA/SFA ratio rises. Moreover, a negative correlation was revealed between retinol and alphatoco levels and TBARS values < −0.6. This inverse relationship suggests that as alpha-tocopherol and retinol levels increase, TBARS values tend to decrease, pointing out a protective effect against oxidation of the substances measured.

## 4. Conclusions

This study examined the effects of drying methods, namely sun-drying, continuous convection drying and ISAD, on the quality of kaddid before and after storage at room temperature for 45 days. ISAD showed efficiency in reducing the effective drying time compared to CCD and SD. All three methods tested revealed a significant effect on the contents of vitamins, TBARS and fatty acids. CCD was effective in improving the lipid oxidation rate of kaddid, expressed in terms of TBARS content. However, for all treatments, TBARS values were below the acceptability threshold of 1 mg MDA/kg recommended for lamb meat. ISAD preserved the highest protein content and the lowest monounsaturated fatty acid content. Storage time did not affect lipid oxidation; however, a decrease in saturated and monounsaturated fatty acids was reported for CCD- and ISAD-dried kaddid. The interaction between the two factors (drying method and storage) did not affect the protein, lipid, or polyunsaturated fatty acid composition of kaddid.

In conclusion, the innovative ISAD drying method has shown interesting results when compared to conventional drying modes, including saving energy due to lower effective drying durations and comparable quality parameters after ambient storage.

## Figures and Tables

**Table 1:** Effect of drying methods and storage time on chemical composition (g/kg DM) of kaddid.

	RM	Storage Time	Mode			Statistics			
			SD	ISAD	CCD	P Mode	P Time	P m × t	SEM
Protein (g/kg DM)	8.07 ^z^	t = 0	6.44 ^w, a^	7.26 ^y, a^	6.55 ^wx, a^	0.0157	0.005	0.082	0.122
		t = 45	6.98 ^wx, b^	7.27 ^x, a^	7.34 ^x, b^				
IMF (g/kg DM)	1.28 ^xy^	t = 0	1.65 ^z, a^	1.20 ^xy, a^	1.50 ^yz, b^	0.0051	0.001	0.66	0.071
		t = 45	1.33 ^xy, b^	0.91 ^w, ab^	1.04 ^wx, a^				

SD: sun-drying; ISAD: interval convective drying; CCD: continuous convective drying; statistical test: linear–mixed model procedure; w, x, y, z: different letters within the same row (different drying mode) differ significantly (*p* < 0.05); a, b: different letters within the same column (different storage days) differ significantly (*p* < 0.05).

**Table 2:** Effects of drying methods and storage time on vitamins (μg/g DM) in kaddid.

	RM	Storage Time	Mode			Statistics			
			SD	ISAD	CCD	P Mode	P Time	P m × t	SEM
α-tocopherol	9.74 ^y^	t = 0	1.56 ^w, a^	7.11 ^x, b^	7.80 ^x, b^	<0.0001	<0.0001	0.0004	0.728
		t = 45	1.19 ^y, a^	2.26 ^xy, a^	3.41 ^x, a^				
ϒ-tocopherol	0.55 ^x^	t = 0	0.26 ^w, a^	0.21 ^w, a^	0.63 ^x, b^	<0.0001	0.17	0.0008	0.034
		t = 45	0.32 ^w, a^	0.29 ^w, a^	0.35 ^w, a^				
δ-tocopherol	0.16 ^z^	t = 0	0.08 ^wx, a^	0.04 ^w, a^	0.13 ^y, b^	0.0048	0.49	0.0005	0.008
		t = 45	0.09 ^y, a^	0.10 ^y, b^	0.09 ^y, a^				
Retinol	0.13 ^x^	t = 0	0.04 ^w, a^	0.12 ^x, a^	0.20 ^y, b^	<0.0001	<0.0001	<0.0001	0.013
		t = 45	0.04 ^w, a^	0.09 ^x, a^	0.05 ^w, a^				

SD: sun-drying; ISAD: interval convective drying; CCD: continuous convective drying; statistical test: linear–mixed model procedure; w, x, y, z: different letters within the same row (different drying mode) differ significantly (*p* < 0.05); a, b: different letters within the same column (different storage days) differ significantly (*p* < 0.05).

**Table 3:** Fatty acids (% of total FAME) of the kaddid.

	RM	Storage Time	Mode			Statistics			
Fatty Acids		SD		ISAD	CCD	P Mode	P Time	P m × t	SEM
C14:0	2.93 ^x^	t = 0	3.42 ^w, a^	3.74 ^w, a^	3.2 ^w, a^	0.044	0.390	0.911	0.09
		t = 45	3.47 ^w, a^	3.94 ^w, a^	3.39 ^w, a^				
C16:0	20.38 ^w^	t = 0	22.54 ^x, a^	23.17 ^x, a^	20.79 ^w, a^	0.003	0.351	0.053	0.22
		t = 45	22.39 ^w, a^	21.81 ^w, a^	21.42 ^w, a^				
C16:1 9c	1.439 ^wx^	t = 0	1.912 ^x, a^	1.34 ^wx, a^	0.87 ^w, a^	0.223	<0.0001	0.0001	0.14
		t = 45	1.90 ^w, a^	1.95 ^w, a^	2.68 ^w, b^				
C18:0	13.93 ^w^	t = 0	11.77 ^w, a^	19.99 ^xy, b^	21.75 ^y, b^	<0.0001	<0.0001	<0.0001	0.78
		t = 45	12.51 ^w, a^	12.38 ^w, a^	11.70 ^w, a^				
C18:1 9c	30.34 ^x^	t = 0	34.22 ^x, a^	29.51 ^wx, a^	25.72 ^w, a^	0.006	0.011	0.012	0.61
		t = 45	33.50 ^w, a^	31.22 ^w, a^	33.15 ^w, b^				
C18:1 11c	0.97 ^w^	t = 0	0.91 ^w, b^	1.24 ^x, b^	1.32 ^x, b^	0.0001	<0.0001	<0.0001	0.06
		t = 45	0.82 ^w, a^	0.85 ^w, a^	0.72 ^w, a^				
C18:2 n6	7.02 ^w^	t = 0	4.89 ^x, a^	3.99 ^w, a^	4.86 ^x, a^	0.582	0.001	0.074	0.15
		t = 45	5.24 ^x, a^	5.63 ^x, b^	5.41 ^x, b^				
C18:3 n3	0.507 ^z^	t = 0	0.339 ^x, a^	0.416 ^y, b^	0.290 ^w, a^	<0.0001	0.07	0.0003	0.009
		t = 45	0.360 ^w, a^	0.373 ^w, a^	0.360 ^w, b^				

SD: sun-drying; ISAD: interval convective drying; CCD: continuous convective drying; Statistical test: linear–mixed model procedure; w, x, y, z: different letters within the same row (different drying mode) differ significantly (*p* < 0.05); a, b: different letters within the same column (different storage days) differ significantly (*p* < 0.05).

**Table 4:** Fatty acid groups (% of total FAME) and ratio of the kaddid.

	RM	Storage Time	Mode			Statistics			
Fatty Acids Groups			SD	ISAD	CCD	P Mode	P Time	P m × t	SEM
SFA	43.31 ^w^	t = 0	44.19 ^w, a^	54.06 ^x, b^	51.88 ^x, b^	0.0005	<0.0001	0.0001	4.45
		t = 45	44.65 ^w, a^	44.38 ^w, a^	43.12 ^w, a^				
MUFA	46.34 ^w^	t = 0	48.39 ^w, a^	38.75 ^x, a^	41.11 ^x, a^	0.001	<0.0001	0.0003	3.92
		t = 45	47.32 ^w, a^	47.53 ^w, b^	48.69 ^w, b^				
PUFA	10.34 ^x^	t = 0	7.42 ^w, a^	7.18 ^w, a^	7.01 ^w, a^	0.940	0.012	0.755	1.22
		t = 45	8.03 ^w, a^	8.09 ^w, a^	8.18 ^w, a^				
PUFA/SFA	0.24 ^x^	t = 0	0.16 ^w, a^	0.13 ^w, a^	0.13 ^w, a^	0.291	0.0002	0.094	0.38
		t = 45	0.18 ^w, a^	0.18 ^w, b^	0.19 ^w, b^				
n6/n3	9.64 ^x^	t = 0	11.60 ^x, a^	6.12 ^w, a^	11.44 ^x, a^	0.002	0.030	<0.0001	0.006
		t = 45	10.33 ^w, a^	11.63 ^w, b^	10.14 ^w, a^				

SD: sun-drying; ISAD: interval convective drying; CCD: continuous convective drying; Statistical test: linear–mixed model procedure; w, x, y, z: different letters within the same row (different drying mode) differ significantly (*p* < 0.05); a, b: different letters within the same column (different storage days) differ significantly (*p* < 0.05).

**Table 5:** Matrix of correlation coefficients calculated between storage time, drying mode, TBARS, α-tocopherols, ϒ-tocopherols, δ-tocopherols, retinol, protein, IMF, and fatty acid groups.

	TBARS	α-toco	ϒ-toco	δ-toco	Retinol	Protein	IMF	SFA	MUFA	PUFA	PUFA/SFA	Storage Time	Mode
TBARS	1.000	−0.692	X	X	−0.681	X	X	X	X	X	X	X	X
α-toco	−0.692	1.000	X	X	0.867	X	X	0.595	−0.668	X	−0.597	−0.503	−0.616
ϒ-toco	X	X	1.000	0.770	X	X	X	X	X	X	X	X	X
δ-toco	X	X	0.770	1.000	X	−0.499	X	X	X	X	X	X	X
retinol	−0.681	0.867	X	0.247	1.000	X	X	0.656	−0.684	X	−0.597	X	−0.647
protein	X	X	X	−0.499	X	1.000	−0.870	X	X	0.525	X	0.568	−0.485
IMF	X	X	X	X	X	−0.870	1.000	X	X	−0.602	−0.516	−0.610	0.616
SFA	X	0.595	X	X	0.656	X	X	1.000	−0.971	X	−0.792	−0.610	X
MUFA	X	−0.668	X	X	−0.684	X	X	−0.971	1.000	X	0.723	0.610	X
PUFA	X	X	X	X	X	0.525	−0.602	X	X	1.000	0.888	0.632	X
PUFA/SFA	X	−0.597	X	X	−0.597	X	−0.516	−0.792	0.723	0.888	1.000	0.744	X
Storage Time	X	−0.503	X	X	X	0.568	−0.610	−0.610	0.610	0.632	0.744	1.000	X
Mode	X	−0.616	X	X	−0.647	−0.485	0.616	X	X	X	X	X	1.000
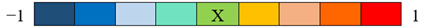													

X: Not significant at 95%; Statistical test: Spearman’s correlation test; Operating parameters: drying mode and storage time: Response variables: TBARS, α-tocopherols, ϒ-tocopherols, δ-tocopherols, retinol, protein, IMF, SFA, MUFA, PUFA, and PUFA/SFA; The values range from −1 (blue) to +1 (red) and indicate statistically significant correlations at the 95% confidence level. The green colour indicates non-significant correlations. Data sharing does not apply to this article.

## Data Availability

Data sharing does not apply to this article.
